# The Impact of the Method Extraction and Different Carrot Variety on the Carotenoid Profile, Total Phenolic Content and Antioxidant Properties of Juices

**DOI:** 10.3390/plants9121759

**Published:** 2020-12-11

**Authors:** Aleksandra Purkiewicz, Joanna Ciborska, Małgorzata Tańska, Agnieszka Narwojsz, Małgorzata Starowicz, Katarzyna E. Przybyłowicz, Tomasz Sawicki

**Affiliations:** 1 Department of Human Nutrition, Faculty of Food Sciences, University of Warmia and Mazury in Olsztyn, Słoneczna 45F, 10-719 Olsztyn, Poland; aleksandra.purkiewicz@student.uwm.edu.pl (A.P.); joanna.ciborska@uwm.edu.pl (J.C.); agnieszka.narwojsz@uwm.edu.pl (A.N.); katarzyna.przybylowicz@uwm.edu.pl (K.E.P.); 2 Chair of Plant Raw Materials Chemistry and Processing, Faculty of Food Sciences, University of Warmia and Mazury in Olsztyn, Pl. Cieszyński 1, 10-726 Olsztyn, Poland; m.tanska@uwm.edu.pl; 3 Institute of Animal Reproduction and Food Research, Polish Academy of Science, Tuwima 10, 10-748 Olsztyn, Poland; m.starowicz@pan.olsztyn.pl

**Keywords:** carrot juices, carotenoids, polyphenols, antioxidant activity, high-speed juicer, low-speed juicer, food processing

## Abstract

The study assesses the antioxidant activity (AA), carotenoid profile and total phenolic content (TPC) of carrot juices obtained from three different varieties (black, orange and yellow) and prepared using high- (HSJ) and low-speed juicer (LSJ). The AA assessment was carried out using four assays (DPPH, ABTS, PCL ACW and PCL ACL). The content of carotenoids was conducted by high performance liquid chromatography equipped with a diode array detector (HPLC-DAD) method, while the total phenolic content by the spectrophotometric method. It was shown that orange carrot juices contain more carotenoids than yellow and black carrot juices, approximately ten and three times more, respectively. The total carotenoid content in orange carrot juice made by the HSJ was higher (by over 11%) compared to juice prepared by the LSJ. The highest total phenolic content was noticed in black carrot juices, while the lowest in orange carrot juices. In black carrot juices, a higher range of TPC was found in juices made by HSJ, while in the case of the orange and yellow carrots, the highest content of TPC was detected in juices prepared by the LSJ. AA of the juices was highly dependent on the carrot variety, juice extraction method. The most assays confirmed the highest AA values in black carrot juices. Furthermore, it was shown that the HSJ method is more preferred to obtain orange and yellow carrot juices with higher antioxidant properties, while the LSJ method is more suitable for black carrot juice extraction.

## 1. Introduction

Carrot is a root vegetable [1] that contains many bioactive compounds, for example, carotenoids (α-carotene, β-carotene lutein, zeaxanthin and lycopene) [2], phenolic acids (chlorogenic, ferulic, p-coumaric, caffeic) [3] and anthocyanins (cyanidin-3-O-xylosyl (sinapoylglucosyl) galactoside, cyanidin-3-O-xylosyl (feruolylglucosyl) galactoside, cyanidin-3-O-xylosyl (coumaroylglucosyl) galactoside) [1]. In addition, carrots are great sources of vitamins (ascorbic acid, thiamine, riboflavin, niacin, pyridoxine, folic acid, vitamin K and A) as well as minerals (calcium, iron, magnesium, phosphorus, potassium, sodium and zinc) [2]. Thanks to the presence of the mentioned bioactive compounds, a carrot has significant health-promoting properties. It was demonstrated that carrots display powerful antioxidant and radical scavenging activities. Moreover, the consumption of carrots has been linked with a lower risk of diseases such as atherosclerosis, cataract, diabetes and cancer [1].

Carrots are commonly classified by the color of roots into white, black, orange, yellow, purple and red [4]. The most common carrot variety is the orange ones which is a genetic crossword between purple, white and yellow carrots [5]. The color of the root has a significant impact on the presence of bioactive compounds. The orange carrot root contains high amounts of α-carotene and is the richest source of β-carotene (precursor of vitamin A). The black carrot is an excellent source of anthocyanins, red carrot root is rich in lycopene, the yellow ones, in turn, was demonstrated to accumulate lutein [6,7]. In addition to the root’s color, the growing and season conditions, the ripeness of carrots as well as part of the root also influence the presence of bioactive compounds [8].

It should be emphasized that the content of bioactive compounds and their biological activity is also influenced by technological processes. However, effect of technological parameters and carrot variety interactions on juice nutrition value is still not fully understood Determination of the composition and content of biologically active substances in both fresh and processed vegetables is crucial for the food industry. Such information is also essential for consumers with an increasingly developing nutritional awareness.

The available literature data show that to date, only a high-speed juicer and traditional blenders were used to examine the impact of the extraction method on the juices’ antioxidant activity. No studies have evaluated the impact of using a high-speed juicer or a low-speed juicer on the antioxidant activity of carrot juices [9,10]. In addition, in the previously published papers, the juices’ antioxidant activity was tested only by DPPH and ABTS assays [9,11,12]. Therefore we intended to examine the influence of the extraction methods on the concentration of main bioactive compounds in the juices obtained from roots of different carrot varieties. To meet this goal we applied low- and high-speed juicer and roots of yellow, orange and black carrots. In addition, to examine the effects of the extraction methods on the antioxidant activity of carrot juices four different methods (DPPH, ABTS, PCL ACW and PCL ACL) were used.

## 2. Results

### 2.1. Content of Carotenoids in Carrot Juices

**Figure 1:**
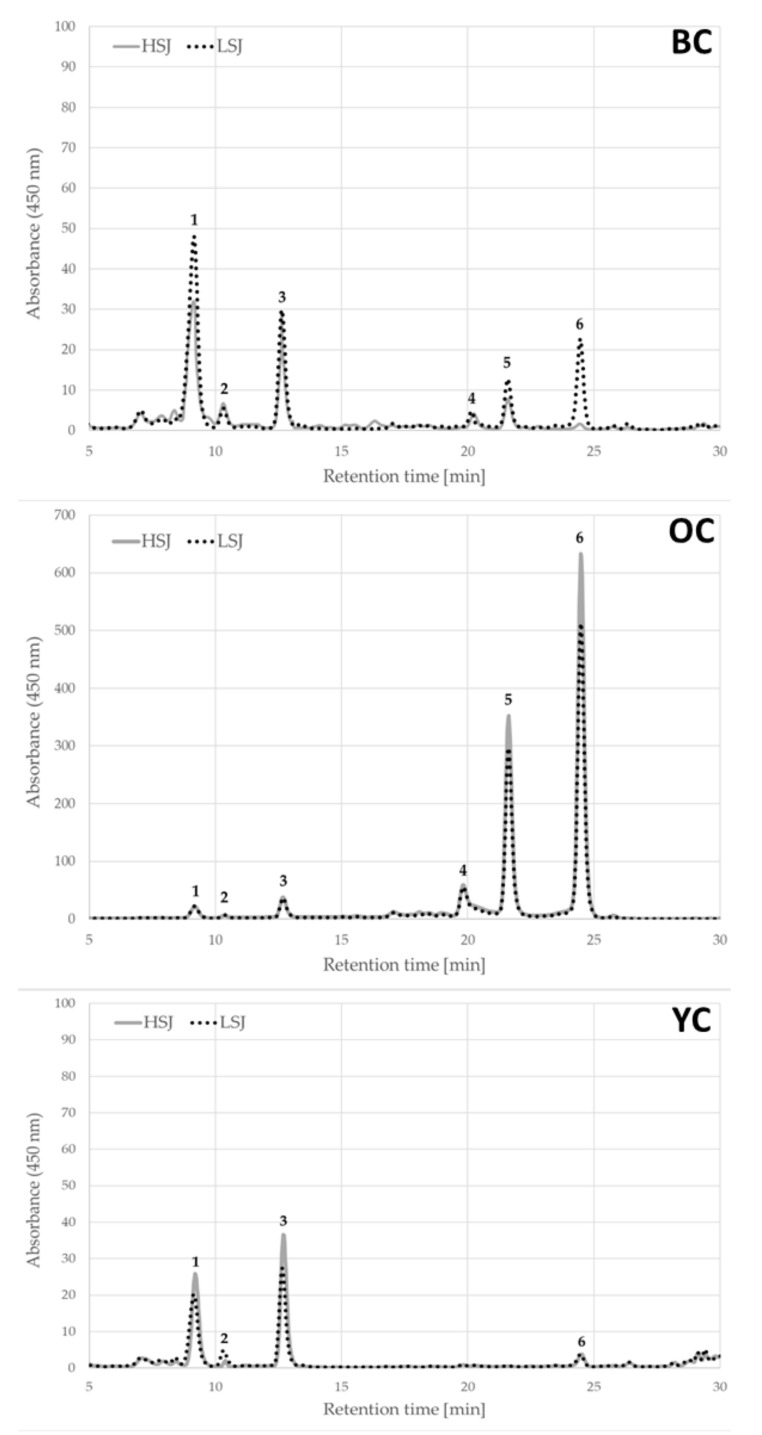
The high performance liquid chromatography (HPLC) chromatograms of carotenoid compounds identified in the carrot juices. Identified peaks are as follows: 1—lutein, 2—zeaxantin, 3—β-apo-8′carotenal (used as an internal standard), 4—13-*cis*-β-carotene, 5—α-carotene and 6—β-carotene. Abbreviations: BC—black carrot, OC—orange carrot, YC—yellow carrot, HSJ—high-speed juicer, LSJ—low-speed juicer.

The results of the total and individual compound content of carotenoids in the tested carrot juices are presented in Table 1. The carotenoid profile in tested carrot juices were clearly more dependent on the carrot variety and this differentiation is visible in Figure 1.

The highest carotenoid content was found in orange carrot juices, while the lowest in yellow ones. The content of t in orange carrot juices was significantly different (*p* < 0.05) from the content in black and yellow carrot juices.

Orange carrot juices contained almost thirty and twenty four times more carotenoids in juices obtained by the use of high-speed juicer and a low-speed juicer than yellow ones and fifteen times (for high speed juicer (HSJ) method) and seven times (for low speed juicer (LSJ) method) more than black carrot juices. The significant difference (*p* < 0.05) was also detected in the carotenoid content of black and yellow carrot juices. Black carrot juices were characterized by two times and over three times more carotenoids content in juices prepared by HSJ and LSJ, respectively, than yellow ones.

In the examined carrot juices five different carotenoids were detected (Table 1). Three compounds belong to the carotenes group (α-carotene, β-carotene and 13-*cis*-β-carotene) and two compounds belong to the xanthophylls group (lutein and zeaxanthin). The richest profile of carotenoids was found in the orange carrot juices. Five compounds (α-carotene, β-carotene, 13-*cis*-β-carotene, lutein and zeaxanthin) were found in the carrot juice obtained by high-speed juicer (HSJ) and by low-speed juicer (LSJ).

The dominant carotenoid in orange carrot juices was β-carotene, constituting 55% and 56% of the total content of carotenoids in juices prepared by the HSJ and LSJ methods, respectively. The second dominant compound, in juices obtained from orange carrots, was 13-*cis*-β-carotene, which was 30% of the total content of carotenoids for juice obtained by the use of HSJ and 32% obtained by the use of LSJ. Juices obtained from yellow and black carrots contained three (lutein, zeaxanthin and β-carotene) and five compounds (lutein, zeaxanthin, β-carotene and 13-*cis*-β-carotene), respectively. In the yellow carrot juices, the dominant carotenoid was lutein which constituted 85% of the total carotenoids content in juices prepared by the use of high-speed juicer and 75% by low-speed juicer. The dominant compounds in the black carrot juices were lutein (63% for HSJ method and 60% for LSJ method) and zeaxanthin (17% for HSJ method and 4% for LSJ method) and 13-*cis*-β-carotene (13% for HSJ method and 11% for LSJ method). Significantly higher amounts (*p* < 0.05) of β-carotene and 13-*cis*-β-carotene were determined in the orange carrot juices compared to the black carrot ones. In turn, the orange carrot juices obtained by HSJ method contained thirty five times more 13-*cis*-β-carotene, whereas juices made by LSJ method contained twenty three times and twenty times more of 13-*cis*-β-carotene and β-carotene, respectively. The highest amounts of lutein were determined in the black carrot juices. The content of this compound was two and fourteen times higher in the black carrot juices than in orange carrot juices made by HSJ and LSJ, respectively. It should be emphasized that, in comparison to other carrot varieties, orange carrot juice contained the lowest percentage of lutein (*p* < 0.05).

It was showed that method of juice extraction significantly affected the content of lutein, zeaxanthin, 13-*cis*-β-carotene and β-carotene in orange carrot juices (*p* < 0.05). Juice prepared by the high-speed juicer contained 77%, 80%, 5% and 8% more these compounds compared to juice obtained using the low-speed juicer (Table 1). Furthermore, the total content of carotenoids in the juice obtained from orange carrots prepared by the HSJ method was significantly higher (by over 111%) than in juice made by the LSJ method (*p* < 0.05).

### 2.2. Total Phenolic Content in Carrot Juices

**Figure 2:**
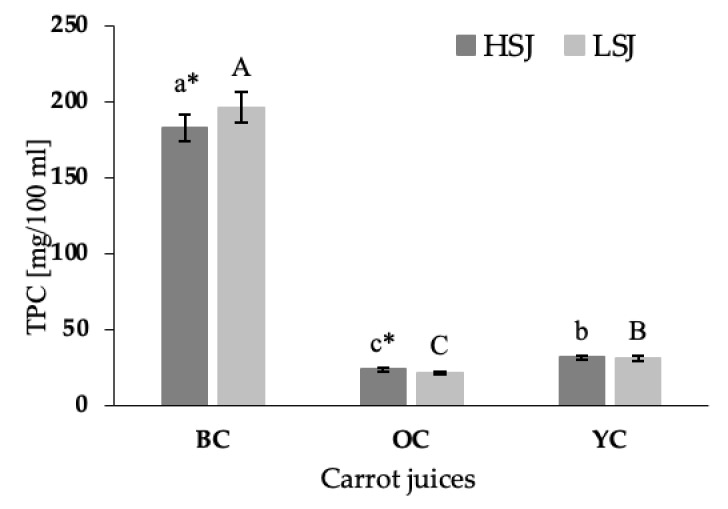
Results of total phenolic content (TPC) in black, orange and yellow carrot juices. Abbreviations: BC—black carrot, OC—orange carrot, YC—yellow carrot, HSJ—high-speed juicer, LSJ—low-speed juicer. Each bar corresponds to the mean of three independent replicates with error bars indicating the standard deviations. Different letters (abc/ABC) indicate significant differences among samples (*p* < 0.05). Statistically significant differences (*p* < 0.05) between method of extraction each carrot variety are marked by *.

The total phenolic content (TPC) in the tested juices differed among carrot varieties (Figure 2). The significantly highest TPC was demonstrated in the juices obtained from black carrots (*p* < 0.05), while the lowest content of these compounds was detected in the orange carrot juices (*p* < 0.05). The black carrot juice prepared with the use of LSJ method contained 7% more total phenolic content than the juice prepared with the use of HSJ (*p* < 0.05). In comparison, the juice obtained from orange carrot using HSJ method was characterized by a higher TPC and contained 10% more these compounds than the juice obtained by LSJ. The TPC demonstrated in the juice obtained from yellow carrot did not differ between the extraction method. The TPC demonstrated in black carrot juices prepared with the HSJ and LSJ methods was eight and nine times higher than TPC demonstrated in orange carrot juices prepared with the same methods, respectively. and six times higher than in yellow carrot juices.

### 2.3. Antioxidant Capacity of Carrot Juices

Four methods (ABTS, DPPH, PCL ACW and PCL ACL) were used to determine the antioxidant activity of the carrot juices (Table 2). The results of the PCL methods were presented both separately and as a sum of ACL and ACE measurements.

The obtained juices were characterized by different antioxidant activity depending on the carrot variety and extraction method. The results of DPPH assay showed that yellow carrot juices demonstrated the highest antioxidant activity among examined varieties (*p* < 0.05) and the juice made by the LSJ method was characterized by slightly higher values of antioxidant activity than those made by the HSJ. In comparison, black and orange carrot juices obtained by the HSJ method were characterized by higher values of antioxidant activity than the juices obtained by the LSJ. A significant relationship (*p* < 0.05) between the extraction method and the antioxidant activity was demonstrated only for black carrot juices (Table 2). The antioxidant activity demonstrated in juice obtained from orange and yellow carrots did not differ between the extraction method (*p* > 0.05).

The results of ABTS assay demonstrated that the highest antioxidant activity was shown for the black carrot juices and the lowest activity was demonstrated for the yellow ones. The antioxidant activity of black carrot juice obtained by HSJ method was 17% and 21% higher than the antioxidant activity of orange and yellow carrot juices, respectively. A similar tendency was observed for the black carrot juices obtained with the use of LSJ. Moreover, the significant relationship between the extraction method and the antioxidant activity estimated by the ABTS test of juices obtained from each carrot variety was noted. The antioxidant activity demonstrated in juices obtained by HSJ method was higher than those shown in juice obtained with the use of LSJ (1.5%-black, 5.5%-orange and 4%-yellow carrot juice) (*p* < 0.05).

The highest antioxidant activity were demonstrated for the black carrot juices, while the lowest values were demonstrated for the orange carrot juices (*p* < 0.05) in all applied assays (PLC ACW, PLC ACL, PLC). The results of PCL ACW assay demonstrated that differences in juices’ antioxidant activity values depends on the carrot variety. The antioxidant activity of black carrot juices method was higher in comparison to the antioxidant activity of orange and yellow carrot juices made (regardless of the extraction method) (*p* < 0.05). It should be noticed that, in case of black and orange carrot, the extraction method had a significant effect on the antioxidant activity determined by all applied assays. The results of all assays showed that the black carrot juice obtained by LSJ had higher antioxidant activity than the juice obtained by HSJ (*p* < 0.05). The opposite situation was observed in case of orange and yellow carrot juices, where the higher antioxidant values were measured in juices obtained by HSJ (Table 2).

### 2.4. Association Between Obtained Data

#### 2.4.1. Linear Pearson’s Correlation Coefficients

The values of antioxidant activity of carrot juices analyzed by the ABTS assay was strongly positively correlated with the values obtained by the PCL ACW (*r* = 0.89), PCL ACL (*r* = 0.96) assays and the sum of PCL assay (*r* = 0.96). While the results of the AC measured by the DPPH assay were only slightly correlated with other assays; negatively with all PCL assays and positively with ABTS assay (Table 3).

Relationships between the content of carotenoids and antioxidant activity were also estimated (Table 3). Statistically significant (*p* < 0.05) and positive correlation coefficients were shown between the content of lutein and the results of ABTS (*r* = 0.91), PCL ACW (*r* = 0.89) and PCL ACL assays (*r* = 0.96) and sum of PCL (*r* = 0.96), while the negative correlation coefficient between the content of lutein and results of the DPPH assay (*r* = −0.91). In turn, average negative correlation coefficients (but not significant at *p* < 0.05) for relationships between content of total carotenoids, α-, β- and 13-*cis*-β-carotene and results of the ABTS, PCL ACW, PCL ACL and PCL assays were noted (r values ranging from −0.29 to −0.51). It was also shown that TPC was highly positively correlated with most applied antioxidant assays; r values in range of 0.79–0.98 for ABTS, PCL ACW, PCL ACL and total PCL assays. However, a strong negative correlation between TPC and DPPH (*r* = −0.83) was found.

#### 2.4.2. Principal Component Analysis (PCA)

**Figure 3:**
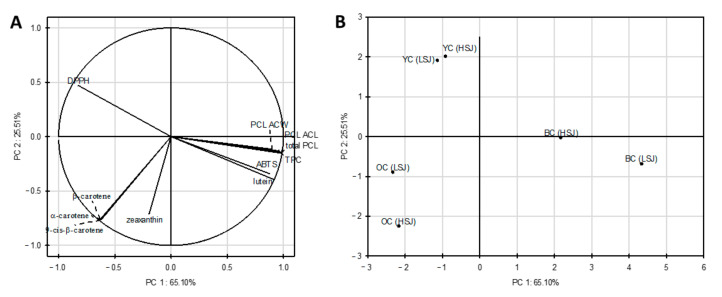
Principal components plot, variations in the parameters (ABTS, DPPH, PCL ACW, PCLACL assays and sum of the PCL) of the analyzed carrot juices (**A**) score plot of the obtained juices (**B**). Abbreviations: BC—black carrot, OC—orange carrot, YC—yellow carrot, HSJ—high-speed juicer, LSJ—low-speed juicer.

Principal component analysis (PCA) was performed on all samples and variables (individual carotenoid concentration, total phenolic content, antioxidant capacity (ABTS, DPPH, PCL ACW, PCL ACL assays and the sum of PCL) to investigate the structure and regularity in the relationships between variables and cases. The first two principal components (PC) explained 90.61% of total data variance. The correlations between the original variables and the obtained principal components are shown in Figure 3a. Each of the variables is represented by a vector. The direction and lengths of the vectors indicate to what extent the given variables affect the principal components. In our study, most input variables are located near the circle, which means that the information in these variables is transferred by principal components. PCA analysis showed a strong positive correlation between α-carotene, β-carotene, 13-*cis*-β-carotene and between lutein and TPC, ABTS, PCL ACW, PCL ACL and the sum of PCL. However, the opposite variables are negatively correlated. The strong negative correlation between lutein and zeaxanthin and the DPPH assay and between ABTS, PCL ACW, PCL ACL, the sum of PCL and DPPH were noted. Moreover, the graph shows that TPC was also negatively correlated with the antioxidant capacity determined by the DPPH assay and positively correlated with the ABTS, PCL ACW, PCL ACL assays and sum of the PCL.

Figure 3b presents the score plot on the plane of principal components, which shows the similarity between the types of carrot juices tested. The analyzed cases’ position concerning each other proves different antioxidant properties of juices obtained from black, orange and yellow carrots. Moreover, based on the analysis, it was observed that the juices from black carrots were the most diverse (LSJ and HSJ methods), while the juices from yellow carrots were the most similar. There was no similarity between the juices from different carrot cultivars.

## 3. Discussion

The carotenoid content in carrot juices varies and depends on many factors, for example, type of raw material and/or storage time [13]. In this study, the highest content of carotenoids was demonstrated in juices made of orange carrot 27.50 mg/100 mL in the juice prepared by the use of HSJ and 24.69 mg/100 mL in the juice obtained by the use of LSJ. In comparison, Amal et al. [13] shown that the total content of carotenoids in the juice from orange carrot was 6.6 mg/100 mL. The difference in the results may arise from the variety of carrot use in the research, growing conditioning and cultivation practice [14]. The lowest carotenoid content was identified in juices from the yellow carrot. Similarly, Sun et al. [15] presented that yellow (as well as white) carrot varieties contain the lowest amount of carotenoid pigments. Therefore, the carrot root color used to juice preparation has an essential contribution to the content of selected compounds from the carotenoid group.

In the conducted research the amount of β-carotene in juices made from orange carrot represented more than 50% of total carotenoids content. In juices made from black carrot the β-carotene content was 20% and in juices from yellow carrot the amount of β-carotene represented 8% (HSJ) and 12 % (LSJ). Juices made of black and yellow carrot contained a higher amount of xanthophylls than carotenes. The group of xanthophylls includes lutein. The available literature data confirms that lutein is the main compound of the yellow carrot and the yellow carrot juices can contain from 0.1 to 0.5 mg/100 mL of lutein [5]. In our experiment, the lutein content in yellow carrot juice was from 0.77 mg/100 mL (LSJ method) to 0.81 mg/100 mL (HSJ method). Black carrot juice possesses 1.5 (from HSJ method) and 2.5 (from LSJ method) times higher content of lutein in comparison to yellow carrot juice. Black carrot juices are considered poor source carotenes but a better source of xanthophylls [5]. More than 79% of carotenoids in juices made of black carrots are zeaxanthin and lutein, which are assigned to the xanthophylls group. Higher content of β-carotene and 13-*cis*-β-carotene was demonstrated in orange juices prepared by the HSJ method and in yellow and black carrot juices prepared by the LSJ method. Similarly to the result obtained in the present study, the result of other researchers demonstrated that the juice preparation method did not significantly influence carotenoid content in juices [11]. On the other hand, the study conducted by Ma et al. [16] showed that the peeling method, blanching and enzyme liquefaction treatment had an impact on β-carotene, α-carotene and lutein contents in the carrot juice. In the case of peeling and blanching, the authors showed a decrease in carotenoid compounds content. On the other hand, use of enzymes in the carrot juice production may significantly increase the content of carotenoids [16].In the present study, the total polyphenols’ content fluctuates in the wide range and depends on carrot variety and juices extraction methods. The highest amount of TPC was observed in black carrot juices. The high content of total phenolic (TPC) in black carrot juices is result of their high concentration in raw material [17]. Total phenolic content in carrot juices determined in the present study was similar to those demonstrated previously by the other authors [18].

The study showed that the extraction method affected phenolics’ content in black and orange carrot juices. In the black carrot juice more phenolics were found in juices prepared with the use of LSJ, while in the orange carrot juices more phenolics contained juices obtained by HSJ. The studies of the other authors also confirm the relationship between the method of juice extraction and the content of polyphenols. For example, the studies of Pyo et al. [9] and Mphahlele et al. [19] demonstrated that juices prepared with the use of blender contain a significantly higher total phenolic content in compared to squeeze juice. Juices made with a blender contain more flesh which is rich in polyphenols, whereas squeezed juices are devoid of pomace to which most polyphenolic compounds pass [9,20].

In presented study, the strong correlations between the content of phenolics and antioxidant capacity measured by ABTS, PCL ACW, PCL ACL and the sum of PCL assays were found. It showed that phenolics play a significant role as antioxidants in carrot juices. The literature data also provided some information on the antioxidant capacity of carrot juices. Kim et al. [12] indicated the higher antioxidant activity of juices from the low-speed juicer. MacDonald-Wicks et al. [21], noticed that antioxidant capacity is the sum of the antioxidant activity of all types of antioxidant compounds present in the product that trap free radicals. In carrots, these compounds include polyphenols, carotenoids and antioxidant vitamins—C and E. The number of values of antioxidant activity of carrot juices for the methods used was as follows: sum of PCL > PCL, ACL > DPPH > PCL, ACW > ABTS. The highest antioxidant activity was established in ABTS, PCL ACW, PCL ACL and the sum of PCL for black carrot juices. According to the literature, methanolic extracts from black carrots possess the highest antioxidant activity determined with the use of DPPH assay [22]. Similarly to the results of present study, the results demonstrated by the other authors showed that, in comparison to juice obtained from yellow and orange carrots, black carrot juices exhibited the highest antioxidants activity [15,22,23,24].

The ratio of antioxidant potential of the lipophilic fraction (ACL) and hydrophilic fraction (ACW) was established at 12.4 and 4.4 in black carrot juices, 21.3 and 20.0 in orange carrot juices and 13.6 and 13.3 in orange carrot juices, obtained by the HSJ and LSJ methods, respectively. These proportions indicated that in a carrot juice, in which hydrophobic antioxidants are predominated, a species-diverse antioxidant profile is presented. The juices’ antiradical activity for fat-soluble compounds (ACL) was significantly higher than the antiradical activity of water-soluble compounds (ACW). The PCL ACL assay indicated antioxidant activity in lipids, in which the soluble carotenoids contained in carrots were found; therefore, a strong positive correlation with the ABTS test was demonstrated. Due to the presence of water-soluble antioxidants in carrot juices (polyphenols and vitamin C) the PCL ACL assay was negatively correlated with the DPPH assay. The strong positive correlation between TPC and the ABTS, PCL ACL, PCL ACW assays and the sum of PCL and the strong negative correlation between TPC and the DPPH assay indicate that polyphenolic compounds that contributed to the increase in the activity of scavenging free oxygen radicals were characterized by different hydrophilicity [22]. In study, the antioxidant activity of carrot juices, measured with the ABTS, PCL ACL, PCL ACW assays and sum of PCL, is positively correlated mostly with the content of lutein, which indicates an important role of this carotenoid in formation of the antioxidant potential of carrot juices.

The juice preparation method had a significant impact on the antioxidant activity of black carrot juices. In the DPPH and ABTS assays, juices obtained with the use of HSJ were characterized by higher antioxidant activity than those obtained with the use of LSJ. In the PCL ACW, PCL ACL and total PCL assays, significantly higher antioxidant activity was confirmed in juices obtained with the use of LSJ method. The highest differences in antioxidant activity in the black carrot juices were demonstrated in the PCL ACW assay and LSJ-obtained juices showed three times higher antioxidant activity than HSJ-obtained juices. In orange carrot juices, in all the performed assays (except DPPH assay), a significant correlation was found between the method of juice extraction and antioxidant activity. In contrast to black carrot juices, in orange carrot juices in each of the assays, higher antioxidant activity was found for juices prepared by the use of HSJ. In yellow carrot juices, only in the ABTS test, there was a significant correlation between the method of juice extraction and the level of antioxidant activity. Higher values of antioxidant activity were obtained in juices prepared with the use of HSJ. In the studies on the influence of the juice extraction method on juices’ antioxidant activity, various relationships were noted—the higher activity of juices prepared with LSJ method [25] or no significant influence of the extraction method on the antioxidant activity of juices [11].

## 4. Materials and Methods

### 4.1. Chemicals and Reagents

2,2′-azinobis(3-ethylbenzothiazoline-6-sulphonic-acid) diammonium salt (ABTS), 2,2-diphenyl-1-picrylhydrazyl (DPPH) and 6-hydrozy-2,5,7,8-tetramethylchroman-2-carboxylic acid (Trolox) were purchased from Sigma Chemical Co. (St. Louis, MO, USA). ACW (hydrophilic condition) and ACL (lipophilic condition) kits for the photochemiluminescence (PCL) assay were received from Analytik Jena AG (Jena, Germany). Hexane, acetone, ethanol, methanol, toluene, sodium thiosulfate, isopropanol, Folin-Ciocalteu reagent, gallic acid, methanol, methylene chloride were purchased from Sigma Chemical Co. (St. Louis, MO, USA). Β-apo-8′-carotenal was received from Sigma-Aldrich (St. Louis, MO, USA).

### 4.2. Plant Material and Juices Extraction

The carrot (*Daucus carota* L.) varieties (Bangor—orange, Gele peen—yellow, Peen zwart—black) were obtained from a local market in Warsaw (Poland). The obtained carrots (5 kg for each variety) were thoroughly cleaned and washed. Carrot extracts were obtained using a low-speed (Kenwood Pure Juice JMP600WH, Warsaw, Poland) and high-speed (Waring Commercial Juice Extractor WJX50, China) juicers. The obtained juices were collected for analysis in appropriately labelled tubes. Carrot extracts were stored at −20 °C until the analysis (up to 2 days).

### 4.3. Determination of Carotenoids Content

The method described by Marx et al. [26] was used to determine the content of carotenoids. Carrot juice sample (5 g) was extracted three times in an amber glass separatory funnel with a 30 mL mixture of acetone and hexane (1:1, *v/v*). The emulsion formed was removed by adding 50 mL sodium chloride solution (10%, *w/v*). After separation, the hexane layer was washed three times with water (50 mL) to remove acetone. Butylated hydroxytoluene (BHT) was added as an antioxidant (0.1%) and the extract was dried with sodium sulfate (2 g). The separated hexane phase was evaporated to dryness under a nitrogen stream at 40 °C and dissolved in 10 mL of methanol-methylene chloride (45:55, *v/v*). The solution was filtered through a membrane filter (ReZist^®^ syringe filter 30 mm, pore size 0.2 μm) and was analyzed by high performance liquid chromatography (HPLC) according to the procedure described by Czaplicki et al. [27]. For analysis it was used an Agilent Technologies 1200 series apparatus (Palo Alto, CA, USA) equipped with a diode array detector (DAD) from the same manufacturer. The separation was carried out on a YMC-C30 150 × 4.6 mm, 3 µm column (YMC-Europe GmbH, Dinslaken, Germany) at 30 °C. The mobile phases consisted of methanol (solvent A) and methyl tert-butyl ether (MTBE) (solvent B) were used. The solvent gradient was as follows: 0–5 min, 95% A, 1 mL/min; 25 min, 72% A, 1.25 mL/min; 33 min, 5% A, 1.25 mL/min; 40–60 min, 95% A, 1 mL/min. The absorbance was measured at the wavelength of 450 nm. Carotenoids were identified based on the retention times of available standards and by comparing the UV-Visible absorption spectra. The content of carotenoids (mg/100 mL) was calculated based on the concentration of the internal standard and expressed in mg/100 mL juice.

### 4.4. Determination of Total Phenolic Content (TPC)

The TPC assay was adapted from the method described by Singleton and Rossi [28]. 0.1 mL of juice sample was mixed with 0.5 mL of Folin-Ciocalteu reagent, diluted 1: 2 with water. Then 1.5 mL of a 14% sodium carbonate solution was added and mixed. The prepared solution was kept in the dark at room temperature for 2 h. Absorbance was measured at 765 nm using an Optizen Pop UV/VIS spectrophotometer (Metasys Co. Ltd., Daejeon, Korea) against a blank sample. The total phenolics content was calculated based on the gallic acid calibration curve (concentrations in the ranges of 10–500 mg/L) and expressed in mg/100 mL juice.

### 4.5. Antioxidant Activity Assays

#### 4.5.1. DPPH Assay

Determination of antioxidant properties by the DPPH method was conducted according to the procedure developed by Brand-Williams et al. [29] and modified by Thaipong et al. [30]. The DPPH radical solution was prepared by dissolving 10 mg of DPPH in 250 mL of 80% methanol. To perform the spectrophotometric test, 300 µL of DPPH solution and 20 µL of juice sample or Trolox solution were mixed. The resulting mixture was left for 30 min at room temperature in the dark. Decreasing absorbance of the resulting solution was monitored at 517 nm using an Infinite M1000 plate reader (Tecan Group AG, Switzerland). The standard curve was determined based on the lag phase’s length compared to Trolox concentrations in the range of 0.01–0.75 mM. The antioxidant activity was expressed as µmol Trolox/mL juice.

#### 4.5.2. ABTS Assay

The ABTS test described by Re et al. [31] was used to determine carrot extract’s antioxidant activity. The measurement required dilution of the ABTS solution using a methanol/water mixture (80:20, *v/v*) to achieve an absorbance level of 0.70 ± 0.02 at 734 nm. For the spectrophotometric test, 290 µL of ABTS solution and 10 µL of the Trolox or juice sample were mixed and absorbance was measured directly after 6 min The standard curve was determined based on the lag phase’s length compared to Trolox concentrations in the range of 0.01–0.75 mM. Measurements were carried out using the Infinite M1000 PRO plate reader (Tecan Group AG, Männedorf, Switzerland). The antioxidant activity was expressed as µmol Trolox/mL juice.

#### 4.5.3. Photochemiluminescence (PCL) Assays

The PCL method with the Photochem apparatus (Analytik Jena, Leipzig, Germany) was used to measure antioxidants’ effectiveness against superoxide anion radicals. Antioxidant activity was analyzed using the ACW (antioxidative capacities of water-soluble) and ACL (antioxidative capacities of lipid-soluble) kits. The assay was conducted as previously described by Sawicki et al. [32]. For ACW and ACL tests, the luminal reagent and Trolox working solution were prepared according to the protocol. Juice solution concentration added that the generated luminescence was in the range limits of the standard curve. Therefore, the lag time (180 s) for the ACW test was used as a free radical scavenging activity. The antioxidant activity was calculated by comparing it with the Trolox standard curve (0.5–3 nmol) and expressed as µmol Trolox/mL juice. In the ACL test, the kinetic light emission curve, showing no lag phase, was monitored for 180 s and expressed in µmol Trolox/mL. The antioxidant test was performed in triplicate for each sample.

### 4.6. Statistical Analysis

The values were expressed as mean ± standard deviation (SD). The results were subjected to a one-way analysis of variation (ANOVA) using Duncan’s test. A linear Pearson’s correlation coefficients were calculated to show relationship between bioactive compounds and antioxidant activity and *p* < 0.05 was considered significant. Principal Component Analysis (PCA) was also carried out to show differences between juice samples. The statistical analysis was performed using Statistica 13.1 (Statsoft Inc., Tulsa, OH, USA).

## 5. Conclusions

The carrot juices obtained in the current study were a rich source of carotenoids and phenolic compounds and the content of these bioactive depended on carrot variety and juice extraction method. It was shown that the orange carrot juices were most abundant in the carotenoids, while black carrot juice was characterized by the highest TPC. The orange carrot juices obtained with the use of HSJ contained a higher amount of carotenoids and phenolic compounds in comparison to the juice obtained using LSJ. Contrary, black carrot juices prepared with the use of HSJ contained significantly lower content of phenolic compounds compared to the juice obtained with the use of LSJ. Moreover, the extraction method had a significant impact on the antioxidant activity of the obtained juices. Among the carrot varieties, the highest antioxidant activity exhibited black carrot juice. The experiments performed with the use of PCL antioxidant activity assays demonstrated that black carrot juice obtained with the use of LSJ was characterized by a higher antioxidant activity in comparison to that obtained with HSJ. Contrary, for orange and yellow carrots juices, a higher antioxidant activity was demonstrated for juices obtained with the use of HSJ. It may be concluded that juices prepared with the use of a low-speed juicer were not always characterized by the higher content of bioactive compounds and antioxidant potential.

## Figures and Tables

**Table 1:** The content (mg/100 mL) of carotenoids in black, orange and yellow carrot juices.

Compounds	Black Carrot		Orange Carrot		Yellow Carrot	
	HSJ	LSJ	HSJ	LSJ	HSJ	LSJ
lutein	1.15 ± 0.49 ^a^	1.98 ± 0.08 ^A^	0.61 ± 0.03 ^c^*	0.14 ± 0.05 ^C^	0.81 ± 0.18 ^b^	0.77 ± 0.02 ^B^
zeaxanthin	0.31 ± 0.06 ^b^	0.14 ± 0.03 ^A^	0.61 ±0.00 ^a^*	0.12 ± 0.03 ^A^	0.06 ± 0.08 ^c^	0.14 ± 0.02 ^A^
α-carotene	0.14 ± 0.05 ^b^	0.16 ± 0.04 ^B^	2.90 ±0.34 ^a^	2.70 ± 0.66 ^A^	ND	ND
13-*cis*-β-carotene	0.24 ± 0.14 ^b^*	0.35 ± 0.03 ^B^	8.30 ± 1.33 ^a^*	7.88 ± 0.08 ^A^	ND	ND
β-carotene	ND	0.68 ± 0.06 ^B^	15.08 ± 2.84 ^a^*	13.85 ± 0.01 ^A^	0.08 ± 0.01 ^b^	0.12 ± 0.02 ^C^
Total	1.84 ± 0.47 ^b^*	3.31 ± 0.79 ^B^	27.50 ± 0.71 ^a^*	24.69 ± 0.51 ^A^	0.95 ± 0.06 ^c^	1.03 ± 0.06 ^C^

Abbreviations: Rt—retention time; HSJ—high-speed juicer; LSJ—low-speed juicer; ND—non-detected. Data are expressed as means ± SD (*n* = 3), means in the line with different letters (abc/ABC) are significantly different (*p* < 0.05). Statistically significant differences (*p* < 0.05) between method of extraction each carrot variety are marked by *.

**Table 2:** The antioxidant activity of black, orange and yellow carrot juices determined by different assays.

Antioxidant Activity Assays [µmol Trolox/mL]	Black Carrot		Orange Carrot		Yellow Carrot	
	HSJ	LSJ	HSJ	LSJ	HSJ	LSJ
DPPH	28.36 ± 0.14 ^c^*	27.66 ± 0.27 ^C^	28.82 ± 0,27 ^b^	28.54 ± 0.35 ^B^	29.08 ± 0.21 ^a^	29.12 ± 0.59 ^A^
ABTS	4.16 ± 0.03 ^a^*	4.10 ± 0.00 ^A^	3.47 ± 0.04 ^b^*	3.28 ± 0.01 ^B^	3.28 ± 0.05 ^c^*	3.15 ± 0.01 ^C^
PCL ACW	9.88 ± 0.17 ^a^*	32.18 ± 0.32 ^A^	4.02 ± 0.03 ^c^*	3.87 ± 0.12 ^C^	6.38 ± 0.32 ^b^	6.28 ± 0.32 ^B^
PCL ACL	122.13 ± 1.73 ^a^*	140.63 ± 5.98 ^A^	85.43 ± 0.46 ^b^*	77.28 ± 0.11 ^C^	86.83 ± 2.16 ^b^	83.35 ± 3.75 ^B^
PCL	132.00 ± 1.56 ^a^*	172.80 ± 5.66 ^A^	89.45 ± 0.43 ^c^*	81.14 ± 0.01 ^C^	93.20 ± 2.47 ^b^	89.63 ± 3.43 ^B^

Abbreviations: HSJ—high-speed juicer; LSJ—low-speed juicer. Data are expressed as means ± SD (*n* = 3), means in the line with different letters (abc/ABC) are significantly different (*p* < 0.05). Statistically significant differences (*p* < 0.05) between method of extraction each carrot variety are marked by *.

**Table 3:** Correlation coefficients (r) calculated for the relationships between carotenoids, total phenolic content and antioxidant activity assays (r marked by * are statistically significant at *p* < 0.05).

Correlation	DPPH	ABTS	PCL ACW	PCL ACL	Sum of PCL
zeaxanthin	0.05	0.18	−0.24	−0.05	−0.11
lutein	−0.91 *	0.91 *	0.89 *	0.96 *	0.96 *
α-carotene	0.09	−0.32	−0.42	−0.51	−0.49
13-*cis*-β-carotene	0.10	−0.31	−0.44	−0.51	−0.50
β-carotene	0.11	−0.33	−0.43	−0.51	−0.50
total carotenoids	0.07	−0.29	−0.40	−0.48	−0.47
TPC	−0.83 *	0.96 *	0.79	0.98 *	0.95 *
DPPH	0.05	0.18	−0.24	−0.05	−0.11
ABTS	−0.91 *	0.91 *	0.89 *	0.96 *	0.96 *
PCL ACW	0.09	−0.32	−0.42	−0.51	−0.49
